# Surgery of the Liver and Gall Bladder

**Published:** 1896-03-21

**Authors:** 


### Progress in Surgery.

### SURGERY OF THE LIVER AND GALL BLADDER.

**Rupture of the Liver** is difficult of diagnosis, and Bryant[1] confesses that there are no special symptoms which can be accepted as indicative of its existence, although severe shock and collapse as immediate results of a forcible local injury to the right side of the abdomen, or to the lower part of the right side of the thorax, should always suggest its probable existence; and even when the same kind of injury to the same regions is unattended by any such serious symptoms as have just been alluded to, the surgeon should be alive to the possibility of a limited rupture of the liver being present, and he should treat the case accordingly. He quotes several cases in support of his argument.

**Suppurating Hydatid of the Liver.** — Paget[2] records two cases which projected backwards, and were drained through the chest wall after resection of a rib. The pleura was laid freely open. There were no pleural adhesions, and the lung was not seen. The diaphragm, which was very tense and pushed up, was stitched to the pleura, so as to shut off the pleural sac, two stitches being put in, one for each edge of the incision through the pleura. The diaphragm between the stitches was incised, the cyst punctured, and then fastened in the wound. The cavity was well washed out, and drained with a very large tube. One case recovered, the other died. The author considers that one should be guided by the indications given by percussion. Where dulness can be clearly made out there the exploring syringe should be put in and thrust well forward. If it draws off pus, then one should go along its track, even though it involves laying the pleura open.

**Cholelithiasis.**—Morison[3] formulates the following: (1) When the gall bladder is distended without jaundice and pain, after the first acute attack is absent, a stone has completely blocked the mouth of the gall bladder or the cystic duct. (2) Attacks of severe pain in the epigastrium and right hypochondrium, accompanied by vomiting and shivering, and followed by sweating, complete relief and transient jaundice, are due to the passage of a gall stone from the gall bladder through the ducts into the duodenum. (3) If the relief after the attack is incomplete, the jaundice more or less persistent, and the patient attacked by ague-like paroxysms, generally with, but perhaps without, pain, each attack being followed by a temporary increase of jaundice, a stone is impacted in, but not completely blocking, the common duct. (4) Persistent jaundice with a distended gall bladder and absence of severe pain are due to a complete block of the common duct, arising usually from malignant disease in its neighbourhood and involving it. (5) Like the urinary bladder, the gall bladder will become contracted and hypertrophied when dealing with a partial obstruction, and like it will dilate painlessly when all its efforts to overcome the obstruction are futile. He lays great stress on the importance of efficient drainage of the pouch[4] which lies beneath the right lobe of the liver, and which is shut off by natural barriers from the general peritoneal cavity. Elliott[5] contends that the operations of cholecystotomy and cholecystenterostomy have become too much the routine practice for the relief of gall stones, and that incision of the ducts or the gall bladder, followed by immediate suture, is the proper operation in the majority of cases. His conclusions are: 1. Every operation should be conducted with the idea of restoring the functions of the ducts, and any irreparable injury to them is a serious calamity. 2. Immediate closure of the gall bladder is safe if the ducts are clear and its walls healthy. 3. Incision and suture of the cystic duct are preferable to prolonged manipulation. 4. Incision and suture of the hepatic and common ducts constitute the operation to be chosen for impacted stones. 5. The mortality of this operation is less than 18 per cent. 6. If the condition of the patient is critical preliminary cholecystotomy is advisable. 7. Cholecystenterostomy should be reserved for irremediable stenosis of the common duct. He reports five successful cases. Kehr,[6] out of 77 cœliotomies for gall stones, found the calculus more or less firmly fixed in the cystic duct in 26 cases. For this latter condition he formerly employed the suture of the gall bladder in the abdominal wound and waited for the pressure of the bile from behind, in conjunction with the diminished swelling of the mucous membrane, to force out the calculus. Now he resolves upon an immediate cystico-lithectomy if he is convinced that the stone will not be spontaneously discharged, if he feels that it is too large to pass through the contracted cystic duct, if the condition of the patient allows further operation, if the cystic duct can be freely exposed so as to render its accurate suture possible, and if an immediate evacuation of the cystic duct is necessary for drainage of the biliary system. Mermann[7] says ideal cholecystotomy or cholecystendysis is the operation of election in cases in which the gall bladder presents but slight changes in its walls, and the contents are bilious, mucous, or serous, as it gives the best results under such circumstances. Cholecystectomy is indicated only in cases of extensive ulceration or malignant degeneration of the gall bladder, on condition, of course, that the ductus choledochus be permeable. When the latter is obliterated by a calculus, which cannot be pushed back into the gall bladder, choledochotomy should be resorted to, whilst cholecystenterostomy is imperative in cases in which the obstruction of the ductus choledochus is not the result of a calculus, or if the direct extraction of a calculus of the choledochus presents too great difficulties. Cholecystostomy in one sitting is the operation of election in all other cases, and should be employed particularly in empyæma of the gall bladder, when the walls of the latter are not the seat of extensive ulceration, and is also called for whenever the gall bladder is comparatively healthy, while the cystic duct is not permeable. Lastly, in cases of malignant jaundice, it may be indicated as a preliminary operation, in order to obviate the danger of cholæmia. Cholecystostomy in two stages is not to be recommended, except in debilitated patients.

**Internal Choledocho-Duodenostomy.** — Kocher[8] says there are many instances in which impacted gall stones can neither be crushed nor pushed one way or the other out of the choledochus. In one case of this class, where the stone was as large as a pigeon's egg, he made a transverse incision on the anterior wall of the duodenum, followed by incisions of the posterior wall of the intestine and the ductus choledochus on the stone, which was pushed forward by an assistant. After extraction of the stone, the edges of the incision into the choledochus were united to the edges of the posterior opening in the duodenum, after which the transverse incision was sutured, and the operation was completed by drainage and partial suturing of the abdominal wound. The patient recovered. The author gives the above name to the operation.

**Angiocholitis and Cholecystitis** are infective in origin according to Terrier,[9] who regards medical treatment as practically useless. Surgeons are agreed that in suppurative angiocholitis surgical operations are called for, but he is inclined to go still further, inasmuch as the cases of non-suppurative angiocholitis and cholecystitis are met with, in which surgical interference is necessary, because the affection, if left to run its own course, terminates in death. The operation which appears to be indicated is cholecystostomy, presenting as it does the following advantages: In the first place, the liquid contained in the gall bladder is drained off, together with the calculi which it may contain; and secondly, the escaping bile removes in a mechanical manner the septic elements present in the bile ducts, so that these become partially disinfected. He quotes three successful cases in support of this opinion.

[1] Lancet, Nov. 9, 1895, p., 1,154. [2] Brit. Med. Journ., Nov. 2, 1895, p. 1,095. [3] Annals of Surgery, Aug., 1895, p. 181. [4] Vide Brit. Med. Journ., Nov. 3, 1894. [5] Brit. Med. Journ., Aug. 10, 1895, and Med. News, June 15, 1895. [6] New York Med. Record, June 15, 1895. [7] Med. Week, Sept. 20, 1895, and Beit. z. Klin. Chir., XIII., 2. [8] Med. Week, Sept. 16, [9] Ibidem, Oct. 25, 1895.

